# Protocol Development for a Qualitative Methodological Study Within a Trial (Qual-SWAT): The KARMA-Dep-2 Trial

**DOI:** 10.12688/hrbopenres.13721.2

**Published:** 2023-11-20

**Authors:** John McCaffrey, Masashi Terao, Cathal McCaffrey, Anna Igoe, Orlaith Loughran, Kelly McDonagh, Sarah McDonogh, Ellie Shackleton, Emma Whooley, Ana Jelovac, Declan M. McLoughlin, Andrew Hunter

**Affiliations:** 1 School of Population Health, RCSI University of Medicine and Health Sciences, Dublin, Ireland; 2 School of Medicine, University of Galway, Galway, Ireland; 3 Department of Psychiatry, Trinity College Dublin, St. Patrick’s University Hospital, Dublin, Ireland; 4 Trinity College Institute of Neuroscience, Trinity College Dublin, Dublin, Ireland; 5 School of Nursing and Midwifery, University of Galway, Galway, Ireland; 6 Qualitative Research in Trials Centre (QUESTS), University of Galway, Galway, Ireland

**Keywords:** mental health, randomised trial, recruitment and retention, study within a trial (SWAT), qualitative, trial methodology

## Abstract

**Background:**

Despite methodological improvements in clinical trial design and conduct more generally, methodological limitations persist in trials concerning mental health care. A qualitative Study Within A Trial (Qual-SWAT), embedded in the KARMA-Dep-2 host trial, will be undertaken to explore and gain an understanding of two methodological questions in randomised trials specific to mental health care: (1) what are the key barriers and enablers of participation in randomised trials in mental health; and (2) how can randomised trials become part of routine mental health care. These issues will be examined from patient-participant and clinician- / researcher-participant perspectives, in alignment with PRioRiTy research themes.

**Methods:**

A descriptive qualitative study design will be used. Data will be collected via one-to-one semi-structured interviews, conducted via Microsoft Teams. The interview data will be analysed using Braun and Clarke’s Thematic Analysis approach. One-to-one interviews will be conducted with three participant groups ( *N* = 30): 1) host trial patient-participants ( *n* = 10); 2) potentially eligible host trial patient-participants who refused enrolment in the host trial ( *n* = 10); and 3) clinician- / researcher-participants who are associated with work on the host trial ( *n* = 10).

**Ethics and dissemination:**

Ethical approval has been granted by St. Patrick’s Mental Health Services Research Ethics Committee, Ireland (Ref: Protocol 09/20). When the study is completed, a report will be prepared and submitted to the Health Research Board (HRB). Findings will be shared with the host trial team and study participants, and submitted for publication.

**Host trial registration:**

ClinicalTrials.gov ( NCT04939649); EudraCT ( 2019-003109-92). Official title: Ketamine as an Adjunctive Therapy for Major Depression - A Randomised Controlled Trial: [KARMA-Dep (2)].

## Introduction

### Background

Despite the increasing improvement and availability of treatments for major depression, prevalence has not decreased concordantly, and depression remains a public health concern (i.e., *treatment-prevalence paradox*; Ormel *et al*., 2022). Debate persists about the ontology of depression and its measurement ( Fried *et al*., 2022; McPherson & Armstrong, 2022), the types of outcome domains assessed in clinical trials and treatment ( Chevance *et al*., 2020; Cuijpers, 2019; Krause *et al*., 2023), and the comparative effectiveness of different treatment modalities on patient outcomes ( Cuijpers *et al*., 2023). Treatment-resistance also presents a complex issue to be addressed when developing clinical research studies and clinical practice guidelines ( Howes *et al*., 2022). A lack of clarity around concepts such as treatment-resistant depression (TRD) and partially responsive depression (PRD) impedes progress in advancing clinical trial design and practice-based recommendations ( Sforzini *et al*., 2022). Identifying methods to improve trial design and efficiency can provide guidance on addressing some of these issues, especially when exploring novel therapeutic approaches ( Correll *et al*., 2023). Germane to this study is the clinical utility of ketamine infusion as an adjunctive therapy for major depression, based in an Irish mental health service ( Gallagher *et al*., 2022).

Randomised controlled trials (RCTs), as they are traditionally designed and conducted, may not be suitable to address the types of research questions and treatment issues that are specific to provision of mental health care ( Chevance *et al*., 2022; McPherson *et al*., 2020). Concerns about the methodological shortcomings in mental health trials go back decades (e.g., Hotopf *et al*., 1997). Methodological limitations can include poor trial recruitment and retention ( Chevance *et al*., 2022; Liu *et al*., 2018), suboptimal sample size, statistical power and reporting bias ( de Vries *et al*., 2023; Vrljičak Davidović *et al*., 2022). Significant research waste and implementation gaps also exist, with limited translation of research findings into practice ( Kazdin, 2022; Minogue *et al*., 2022; Yordanov *et al*., 2015).

One approach to improving trial design, conduct, and recruitment is the use of qualitative methodologies ( Hennessy *et al*., 2018; Kazdin, 2022; Powell *et al*., 2022; Tan *et al*., 2022). A Cochrane synthesis of qualitative studies considered factors that influence decision-making regarding trial participation, including the manner in which trial information is communicated to potential participants, how the trial is designed, how participant decision-making may be influenced by others (e.g., recruiters), and how risk-benefit perceptions and communication impact recruitment ( Houghton *et al*., 2020). According to a recent *Lancet Psychiatry* review, only a relatively few qualitative research studies “have identified the barriers and facilitators for either patient participation in mental health RCTs or participant recruitment by clinicians” ( Chevance *et al*., 2022). Although research has identified both positive and negative impacts of service user engagement in research ( Happell *et al*., 2018; McCabe *et al*., 2023; Newmark *et al*., 2020; Russell *et al*., 2020; Staley *et al*., 2013), there is a lack of research exploring the impacts of involving mental health service users in the design and conduct of clinical trials. In addition, research is needed to identify strategies on how best to engage clinicians / healthcare professionals in conducting research activities ( Yoong *et al*., 2023).

A promising approach to address this gap, to improve trial design and conduct, and reduce research waste, is the use of Study Within A Trail (SWAT) methodology ( Ahmed *et al*., 2022; Allan *et al*., 2021; Boxall *et al*., 2022; Thiblin *et al*., 2022). A SWAT can be defined as a “self-contained research study that has been embedded within a host trial with the aim of evaluating or exploring alternative ways of delivering or organising a particular trial process” ( Treweek *et al*., 2018). Challenges to implementing SWAT protocols within host trials have been noted, including a lack of funding and understanding about SWAT methodology, perceptions that a SWAT will add to the complexity of an already complex study process, and concerns about the increased burden placed on research participants ( Arundel *et al*., 2023; Clark *et al*., 2022; Doherty *et al*., 2023). Additional research is needed to explore the use of SWAT methodology in clinical research generally, and in mental health research specifically.

### Study aims and objectives

Currently, there is a lack of research knowledge about the feasibility and utility of using qualitative SWAT methods to explore randomised trial processes in mental health care, and this study aims to address this gap. This qualitative Study Within A Trial (Qual-SWAT) will be embedded in the KARMA-Dep-2 Trial (Ketamine as an Adjunctive Therapy for Major Depression), to address two key methodological questions relating to the design and conduct of trials as part of mental health service provision. The methodological questions proposed arise from the PRioRiTy studies (Prioritising Recruitment / Retention in Randomised Trials), which identified a list of the top ten priority research questions to be addressed concerning recruitment and retention in randomised trials ( Brunsdon *et al*., 2019; Healy *et al*., 2018). The specific methodological questions guiding the development of this study are as follows:

1. How can randomised trials become part of routine mental health care and best utilise current clinical care pathways?
2. What are the barriers and enablers for clinicians / healthcare professionals in helping conduct randomised trials?

The purpose of this study is to identify the key barriers and facilitators encountered when participating in a randomised mental health trial, from a patient-participant perspective, and to gain an understanding of how randomised trials can become part of routine mental health care. This study also aims to identify key barriers and enablers to recruitment and retention in mental health trials, from a clinician perspective, in order to guide future delivery, education and research. In addition, the study findings will be fed back to the host trial research team, to provide support for ongoing recruitment and retention within the host trial. A key objective, using both qualitative and SWAT methodology, is to contribute to the rigorous development of a participant-led understanding of the two identified PRioRiTy methodological questions. By exploring the perspectives of patients and healthcare professionals, it may be possible to develop strategies to prevent inefficiency and waste in clinical trials ( Gillies *et al*., 2019).

## Methods

### Ethics

This study will adhere to the Declaration of Helsinki ( World Medical Association, 2013). Ethical approval for the host trial was granted by the Mater Misericordiae Institutional Review Board (Ref: 1/478/97) and St. Patrick’s Mental Health Services Research Ethics Committee (Ref: 05/20) and for the Qual-SWAT study (Ref: Protocol 09/20). Participants will be informed of their right to withdraw from the study at any stage without penalty or consequence. As the sample will include psychiatric patients, it is important to acknowledge that they will only be included in this study if they have the ability and capacity to give fully informed consent and are stable. The research team has developed a distress protocol for the conduct of interviews, and this will be strictly adhered to during the conduct of all interviews ( Draucker *et al*., 2009; Pinto *et al*., 2022; Whitney & Evered, 2022). A period of time will be given to all participants post-interview, to discuss any issues relating to the interview process that may have caused potential distress, and support will be offered if necessary.

### Host trial

The host study is a pragmatic, randomised controlled, parallel-group, superiority trial, which is investigating the use of ketamine as an adjunctive therapy for major depression (KARMA-Dep-2: ClinicalTrials.gov NCT04939649; EudraCT 2019-003109-92).

### Study design

This is a qualitative, Study Within A Trial (SWAT), embedded in the KARMA-Dep-2 trial. This qualitative SWAT (Qual-SWAT) is conducted independently from the host trial. This study will use a descriptive qualitative design, relying on purposive sampling. One-to-one semi-structured interviews will be conducted via Voice over Internet Protocol technology (VoIP; Tomás & Bidet, 2023), using Microsoft Teams.

### Study participants and procedure

According to a systematic review, a sample size of 9 - 17 interviews is sufficient to achieve saturation, which is a cornerstone of rigour in qualitative research ( Hennink & Kaiser, 2022). Three groups of participants will be invited to take part in qualitative interviews ( *N* = 30). These sub-groups include: (1) participants who agree to participate in the host trial ( *n* = 10); (2) participants who are invited to take part in the host trial and decline participation ( *n* = 10); and (3) clinicians and researchers who are associated with work on the host trial ( *n* = 10). To be eligible for inclusion in the Qual-SWAT, participants must be: (1) over the age of 18 years with the ability to give fully informed consent; (2) been deemed eligible for participation in the host trial; and (3) a clinician or researcher working on the host trial. For details on the host trial inclusion/exclusion criteria, see the trial registration (ClinicalTrials.gov NCT04939649). Clinicians and researchers who are not associated with the host trial will be excluded as potential participants in the Qual-SWAT.

Recruitment for the Qual-SWAT will take place at two clinical settings across St. Patrick’s Mental Health Services (i.e., St. Patrick’s University Hospital, Dublin, and St. Patrick’s Hospital, Lucan). Potential participants are pre-screened for eligibility by reviewing electronic health records of all physical hospital admissions. Eligibility is determined by a host trial investigator who is a medical doctor. Potential participants are approached within 10 days of admission by a member of the research team and given information about the study (i.e., participant information leaflet). The host trial investigators act as gatekeepers, by identifying potential participants and distributing study information packs. If agreeable to participate, written informed consent is obtained by host trial staff. The host trial consent form provides an option for participants to agree to future contact for follow-up research studies that may arise from the host trial.

Potential participants will be given sufficient time; to allow for a period of time to review and consider the study information provided, to ask any questions they have, and to make a fully informed decision with regards to their participation in the study. Following the reflection period, arrangements will be made to proceed with the research study with those who have indicated an interest in participating. All participants in the Qual-SWAT will be asked by a trial investigator to read and return a signed consent form before undertaking interview.

### Interview guide

The interview guide will include questions about the experience of trial recruitment, key issues relating to participation, views on trial conduct, processes, and integration of research into day-to-day care, and views on further research in mental health care settings ( Table 1). In addition, observational and field notes will be included during the interview process.

**Table 1.** Interview guide.

**Interview Guide**	
**Opening and introduction:** The aim of this interview is to further understand your experience, opinions and perceptions regarding recruitment to the **KARMA-Dep (2)** **Trial.**The interviewer should explain that this is a guided discussion to ascertain the participant’s experience. **We are specifically looking for data on: why you chose to participate / decline, experience ** **of participation and views on trial conduct and integration of research in day to day care.**	
**Discussion Topic Guide**	
**Questions**	**Notes**
**Experience of Recruitment**	
What is your experience of being recruited to the trial?	
Why did you provide consent / refuse to participate in the trial?	
What do you know about the process of recruitment for this trial? How do you think it can be improved?	
**Important issues in relation to Participation?**	
What issues are important to you in relation to being involved in the trial?	
How did your understanding of research in inpatient settings change following agreeing to participate?	
**Possible changes in Trial Process?**	
What is the impact of participation on you personally? Professionally?	
How does participation in this trial impact on your well-being? View of trial conduct?	
**Views on further research in Mental Health Inpatient Settings**	
Did you find that the clinical staff on the ward understood and supported the research process?	
Are there any ways to make participating in research while in hospital easier for future studies?	
Are you happy for us to contact you about future research?	
Is there anything else that you would like to add that you think is important about your experience and/or needs?	
● Thank everyone for participating.	

### Analysis and rigour

Demographic information will be collected in order to describe sample characteristics. SPSS will be used to manage quantitative data and report on sample descriptive statistics. The Microsoft Teams interviews will be recorded and transcribed verbatim, and then analysed to determine common themes. Thematic analysis will be used to analyse interview data, using a six-stage approach ( Braun & Clarke, 2006). This involves listening to recordings, reading and rereading the transcripts, coding the data, categorising the codes and finally developing themes. A computer-assisted qualitative data analysis software package (CAQDAS; e.g. NVivo) will be used to aid qualitative data management and analysis ( Clarke *et al*., 2021; Dalkin *et al*., 2021; Kalpokas & Radivojevic, 2022). Data analysis will be undertaken by the Qualitative Research in Trials Centre (QUESTS; https://www.hrb-tmrn.ie/research-innovation/qualitative-research-in-trials/) team, who will familiarise themselves with the data, identify a thematic framework and undertake analysis to produce summary statements explaining the main themes emerging from the data.

Various methods will be used to enhance rigour, based on recommended good practice ( Forero *et al*., 2018; Nowell *et al*., 2017; Patterson *et al*., 2023; Yadav, 2022). Data will be sourced from multiple participant groups to capture a range of perspectives and enhance data completeness (i.e., *triangulation*). NVivo functions (e.g., annotation) will be used to facilitate transparent decision-making processes within and between researchers (i.e., *audit trail*). Two researchers will complete an independent initial analysis of one transcript, and then discuss, compare and agree on initial themes (i.e., *inter-coder reliability*). Agreed themes will be discussed by the entire research team to enable ongoing reflexive analysis. The Standards for Reporting Qualitative Research will be used to guide reporting (SRQR; O’Brien *et al*., 2014).

### Data management

This study will operate in accordance with the Data Protection Act (2018), General Data Protection Regulation (2018) and Health Research Regulations (2018) ( Clarke *et al*., 2019; Mee *et al*., 2021). Participant confidentiality and anonymity will be maintained throughout the research process. Participant coding will occur on recruitment. Data pseudonymisation will be applied throughout data collection and analysis phases, with code numbers applied to all digital audio recordings and transcripts. All electronic data will be password protected and stored securely in accordance with the Data Protection Act (2018).

### Dissemination

A knowledge exchange and dissemination plan will be prepared and agreed with the PI of the host trial. In consultation with the Health Research Board (HRB), a written report will be submitted to the HRB. The report will be made available to the host trial team and to research study participants. Findings will be disseminated and presented at national and international conferences, and submitted for publication to a peer-reviewed journal.

## Conclusion

There is limited research exploring the impacts of involving mental health service users in the design and conduct of clinical trials. Research is also needed to identify strategies on how to engage clinicians / healthcare professionals in conducting clinical research activities, and how best to embed clinical trials into routine clinical practice. This Qual-SWAT aims to add to the evidence base, specifically by developing an understanding of the potential benefits to participation in mental health trials, as well as barriers to recruitment and retention.

### Study status

Following substantial COVID-19 and post-pandemic delays in recruitment, interviews are ongoing. To date, 20 participants have been recruited to this Qual-SWAT study.

## Data Availability

No data are associated with this article.
