# Supplementary material for: Protocol Development for a Qualitative Methodological Study Within a Trial (Qual-SWAT): The KARMA-Dep-2 Trial
Source: HRB Open Res. 2023 Nov 20;6:29. Originally published 2023 May 10. [Version 2] doi: 10.12688/hrbopenres.13721.2 (PMC10285324; doi:10.12688/hrbopenres.13721.2)
Supplement: Peer review history

# Reviewer report: Reviewer response for version 2

**DOI:** 10.21956/hrbopenres.15105.r37095

**Published:** 2023-11-22

**Authors:** John A. King

**Affiliations:** 1 Department of Clinical, Educational and Health Psychology, University College London, London, UK

No further comments.

Is the study design appropriate for the research question?

Yes

Is the rationale for, and objectives of, the study clearly described?

Yes

Are sufficient details of the methods provided to allow replication by others?

Yes

Are the datasets clearly presented in a useable and accessible format?

Not applicable

Reviewer Expertise:

Mental health, psychotherapy, clinical trials, neuroscience, virtual reality, brain imaging, electrophysiology, PTSD, depression, compassion.

I confirm that I have read this submission and believe that I have an appropriate level of expertise to confirm that it is of an acceptable scientific standard.

# Reviewer report: Reviewer response for version 1

**DOI:** 10.21956/hrbopenres.15008.r33992

**Published:** 2023-06-20

**Authors:** Emily Taylor

**Affiliations:** 1 Department of Clinical and Health Psychology, The University of Edinburgh, Edinburgh, UK

This is a clearly written protocol for a well-thought through and valuable study that makes good use of a pre-existing trial. My comments below are caveated by the fact that the study is already underway.

 The protocol requires a long period between invitation to participate and consent and participation, which might result in lost participants, especially in the sub-sample who have exited the host study. Reading on I was a little unclear as to whether this is a minimum or maximum time, but either way might be restrictive. Can you clarify what the 14 days refers to? If it is a minimum requirement and there is scope to reduce this, I would recommend doing so.

 The multi-perspective approach will be valuable in this study. The sample size is ambitious but is presumably feasible in the context of the host study. If sub-samples are seriously unbalanced, how problematic would this be, and have the authors set some lower limits for the sub-samples to ensure that all parties are properly represented?

 Is there an option to interview in-person as an alternative to Teams? Some participants may prefer this.

Is the study design appropriate for the research question?

Yes

Is the rationale for, and objectives of, the study clearly described?

Yes

Are sufficient details of the methods provided to allow replication by others?

Yes

Are the datasets clearly presented in a useable and accessible format?

Not applicable

Reviewer Expertise:

qualitative designs and trials in developmental trauma with care-experienced children and young people

I confirm that I have read this submission and believe that I have an appropriate level of expertise to confirm that it is of an acceptable scientific standard.

# Response

**Published:** 2023-11-16

**Authors:** John McCaffrey

**Affiliations:** RCSI University of Medicine and Health Sciences, Ireland

We thank the reviewer for their insightful and helpful comments.

 Regarding the 14-day specifier, we have updated the text, and rephrased as follows: “Potential participants will be given sufficient time; to allow for a period of time to review and consider the study information provided, to ask any questions they have, and to make a fully informed decision with regards to their participation in the study. Following the reflection period, arrangements will be made to proceed with the research study with those who have indicated an interest in participating”. This wording reflects the practicalities of timing and access in the acute clinical setting, while ensuring that potential participants have time to make an informed decision.

 Regarding sample size, we have adjusted the recruitment plan, and updated the text as follows: “Three groups of participants will be invited to take part in qualitative interviews ( *N* = 30). These sub-groups include: (1) participants who agree to participate in the host trial ( *n* = 10); (2) participants who are invited to take part in the host trial and decline participation ( *n* = 10); and (3) clinicians and researchers who are associated with work on the host trial ( *n* = 10)”. These changes reflect the host trials difficulties in recruiting participants and the changes in sample size have been approved by the ethics committee.

 Regarding interviewing participants in-person, all potential participants are given the option of telephone, Teams or face-to-face if neither of these suits. No participants indicated face-to-face.

# Reviewer report: Reviewer response for version 1

**DOI:** 10.21956/hrbopenres.15008.r33991

**Published:** 2023-06-16

**Authors:** John A. King

**Affiliations:** 1 Department of Clinical, Educational and Health Psychology, University College London, London, UK

The proposal is carefully written and makes the case very well, I firmly support the progress of the project. The use of qualitative research methods within a quantitative superiority RCT is of particular value in addressing well-known concerns about the potentially reductive nature of assessing mental health outcomes numerically. The choice of qualitative approach, thematic analysis, is appropriate for a sample of this size, and the formulation of the questionnaire appears to address the research questions well. The only concern I had, which is not easily addressed and not at all unique to this project, is the inevitable sample bias in trying to interview people who have already declined to take part in the larger study. However in this case I suspect declining will have been to do with not wanting to take ketamine rather than absolutely not wanting to participate in research, and getting hold of a sample could be reasonably straightforward. Additionally, understanding this group will be of particular value, especially if the host trial indicates effectiveness of ketamine.

 Overall this is a strong proposal and in my view should be supported.

Is the study design appropriate for the research question?

Yes

Is the rationale for, and objectives of, the study clearly described?

Yes

Are sufficient details of the methods provided to allow replication by others?

Yes

Are the datasets clearly presented in a useable and accessible format?

Not applicable

Reviewer Expertise:

Mental health, psychotherapy, clinical trials, neuroscience, virtual reality, brain imaging, electrophysiology, PTSD, depression, compassion.

I confirm that I have read this submission and believe that I have an appropriate level of expertise to confirm that it is of an acceptable scientific standard.

# Response

**Published:** 2023-11-16

**Authors:** John McCaffrey

**Affiliations:** RCSI University of Medicine and Health Sciences, Ireland

We thank the reviewer for their helpful comments.

 On the issue of sample bias, initial contact with potential participants who refused the main trial confirms exactly the reviewer’s suggestion that refusal relates to stigma regarding ketamine or wariness of engaging in infusions along with not wanting to interfere with treatment as normal. Understanding these motivations will be a key element of analysis.
